# AI-Powered Marine Drug Discovery: A Putative Dual c-Met/VEGFR2 Lead Candidate for Hepatocellular Carcinoma via Deep Learning and Multiscale Simulation

**DOI:** 10.3390/cimb48090902

**Published:** 2026-09-03

**Authors:** Ruiqi Zhao, Yuhan Wang, Mengyao Han, Jiesheng Guo, Hui Hu, Shiqi Tang, Mengqing Ma, Xiaozhou Zhou, Jialing Sun

**Affiliations:** 1 The Fourth Clinical Medical College, Guangzhou University of Chinese Medicine, Shenzhen 518033, China; 20252120304@stu.gzucm.edu.cn (R.Z.);; 2 The Second Clinical Medical College, Guangzhou University of Chinese Medicine, Guangzhou 510006, China; 3 Faculty of Chinese Medicine, Macau University of Science and Technology, Taipa, Macao 999078, China

**Keywords:** marine drug discovery, artificial intelligence, deep learning, hepatocellular carcinoma, c-Met, VEGFR2, natural products, virtual screening

## Abstract

Background: Hepatocellular carcinoma (HCC) remains a leading cause of cancer mortality. The c-Met and VEGFR2 pathways synergistically drive HCC progression. Marine natural products offer chemically diverse drug reservoirs; however, conventional activity-guided isolation faces labor intensity, low throughput, and frequent compound rediscovery, limiting marine drug development. Objective: To pioneer an artificial intelligence-driven marine drug discovery workflow integrating deep learning virtual screening for identifying dual c-Met/VEGFR2 promising in silico candidate from marine natural product repositories. Methods: UniSite predicted binding pockets in c-Met (PDB: 4R1V) and VEGFR2 (PDB: 2XIR). Drug-likeness filtering of 695,000 compounds from COCONUT and CMNPD databases yielded 84,730 candidates. DiffDock-based screening identified dual-target binders, validated through 200 ns molecular dynamics simulations, MM-GBSA calculations, and DFT analyses. Results: The marine phthalide CMNPD30506 [(S)-3-ethyl-5,6-dihydroxyphthalide] emerged as the lead candidate, engaging VEGFR2 via four hydrophobic contacts and one π-cation interaction with LYS868, while binding c-Met through four hydrophobic interactions, two hydrogen bonds, and π-π stacking. Molecular dynamics demonstrated stable RMSD profiles and dynamic hydrogen bond enrichment. MM-GBSA revealed binding free energies of −14.79 and −13.28 kcal/mol for VEGFR2 and c-Met, respectively, driven by van der Waals forces. DFT calculations indicated a HOMO-LUMO gap of 2.410 eV. Conclusions: This AI-augmented workflow successfully identified CMNPD30506 as a promising dual c-Met/VEGFR2 HCC therapeutic from marine libraries, overcoming traditional discovery bottlenecks through integrated deep learning and physics-based simulations, exemplifying AI’s potential in marine pharmacological research.

## 1. Introduction

Hepatocellular carcinoma (HCC), the predominant histological subtype of primary liver malignancies, accounts for over 90% of liver cancer diagnoses and ranks as the sixth most prevalent neoplasm globally while representing the third leading cause of cancer-related mortality [1]. According to global cancer epidemiological data, approximately 870,000 new HCC cases and 760,000 deaths were documented worldwide in 2022 [2]. Despite incremental advances in early diagnostic modalities and therapeutic interventions, the majority of patients present with intermediate-to-advanced disease at initial diagnosis, compounded by underlying hepatic cirrhosis, culminating in 5-year survival rates below 20% [3]. Currently, multi-targeted tyrosine kinase inhibitors (TKIs) including sorafenib, lenvatinib, and cabozantinib have garnered regulatory approval for systemic management of advanced HCC; however, clinical efficacy remains modest, with median overall survival extensions of merely 2–3 months accompanied by considerable adverse event profiles [4,5]. Consequently, the development of novel, highly efficacious, and minimally toxic HCC therapeutics constitutes a pressing imperative in contemporary oncological research. The receptor tyrosine kinase c-Met (hepatocyte growth factor receptor) occupies a pivotal position in HCC pathogenesis and progression [6]. Aberrant overexpression or constitutive activation of c-Met and its cognate ligand hepatocyte growth factor (HGF) manifests in approximately 40% of HCC patients, correlating intimately with tumor proliferation, invasion, metastatic dissemination, and unfavorable clinical outcomes [7,8,9]. Activation of the HGF/c-Met signaling axis potentiates downstream RAS/MAPK and PI3K/AKT pathway engagement, thereby orchestrating tumor cell survival, epithelial-mesenchymal transition (EMT), and neovascularization. Experimental evidence substantiates that c-Met pathway inhibition effectively suppresses HCC cellular proliferative and migratory capacities [10]. Concurrently, vascular endothelial growth factor receptor 2 (VEGFR2) serves as a master regulator of tumor angiogenesis, demonstrating elevated expression in HCC tissues and promoting neovascular formation via the VEGF/VEGFR2 signaling conduit, thereby furnishing nutrient and oxygen supplies essential for tumor expansion [11,12]. Critically, intricate crosstalk and compensatory mechanisms exist between the c-Met and VEGFR2 pathways. Selective VEGFR2 inhibition precipitates tumor hypoxia, consequently upregulating hypoxia-inducible factor-1α (HIF-1α) and triggering compensatory c-Met pathway activation as an escape mechanism, ultimately engendering therapeutic resistance [13].

Predicated upon these mechanistic insights, dual-targeting strategies simultaneously engaging c-Met and VEGFR2 theoretically enable synergistic antitumor efficacy while circumventing monotherapy resistance. This conceptual framework has garnered clinical validation: cabozantinib, a dual c-Met/VEGFR2 inhibitor, received FDA approval for second-line HCC treatment following sorafenib failure, demonstrating significant improvements in overall survival and progression-free survival [14,15]. Nevertheless, existing dual-target inhibitors remain encumbered by insufficient selectivity, off-target effects, and toxicity liabilities [16], thereby establishing the clinical imperative for next-generation, highly selective, minimally toxic c-Met/VEGFR2 dual inhibitors.

Natural products constitute a historically rich reservoir for drug discovery, conferring unique advantages in anticancer pharmaceutical development. Statistical analyses indicate that approximately 50% of approved anticancer agents derive directly from natural products or their structural analogs [17]. Natural products characteristically exhibit high structural diversity, potent biological activities, and comparatively attenuated toxicity profiles [18]. Marine natural products, in particular, owing to distinctive ecological selection pressures, have evolved chemical scaffolds absent in terrestrial organisms, thereby providing abundant chemical diversity for pharmaceutical innovation [19].

While marine organisms have yielded therapeutics—including the antitumor agents trabectedin and eribulin—the discovery process has historically relied on bioactivity-guided fractionation, a labor-intensive approach requiring iterative extraction, chromatographic separation, and bioassay testing of crude extracts. This canonical strategy, though successful, faces inherent limitations: (1) low throughput due to manual processing bottlenecks; (2) high rediscovery rates of abundant “nuisance compounds” masking rare bioactive entities; (3) supply constraints from marine organism collection and culture; and (4) limited chemical space exploration restricted to readily extractable metabolites. These challenges have contributed to declining productivity in marine natural product drug discovery despite the ocean’s estimated chemical diversity exceeding terrestrial sources by orders of magnitude.

In recent years, the rapid evolution of computer-aided drug design (CADD) technologies has furnished efficient and economically viable solutions for pharmaceutical development [20]. Propelled by unprecedented advances in artificial intelligence, applications of AI in drug discovery (AIDD) are experiencing exponential growth, with AIDD poised to fundamentally transform and revolutionize traditional drug development paradigms in the foreseeable future [21,22]. Virtual screening, as a cornerstone AIDD technology, enables rapid identification of bioactive molecular entities from large-scale compound libraries, substantially abbreviating drug discovery timelines while mitigating research and development expenditures. Particularly noteworthy is the application of deep learning architectures in pharmaceutical discovery, exemplified by deep neural network-based molecular docking platforms such as DiffDock, which exhibit marked improvements in both accuracy and computational efficiency relative to conventional methodologies [23].

Despite this promise, AI-driven marine drug discovery remains underexplored. Most AI pharmaceutical applications focus on synthetic libraries or terrestrial natural products. This gap persists partly due to marine natural product databases’ historical fragmentation and incomplete bioactivity annotations. The recent consolidation of open-access marine databases, The Comprehensive Marine Natural Products Database (CMNPD), housing roughly 47,000 marine-derived compounds accompanied by structured bioactivity data and taxonomic organization, currently offers essential data architecture supporting rigorous AI model development and implementation [24]. Leveraging these resources through integrated AI pipelines represents a frontier opportunity to revitalize marine drug discovery productivity.

In synthesis, this investigation aimed to integrate state-of-the-art AI-assisted virtual screening technologies with multiscale computational simulation methodologies to systematically identify lead compounds exhibiting dual c-Met/VEGFR2 inhibitory potential from extensive natural product libraries.

## 2. Materials and Methods

### 2.1. Retrieval and Processing of Target Protein Architectures

Atomic-resolution structural coordinates for proteins of interest were obtained from the RCSB Protein Data Bank (PDB) database (https://www.rcsb.org/), with VEGFR2 (VEGFR2) represented by PDB ID 2XIR and c-Met by PDB ID 4R1V. Downloaded PDB files were subjected to visual inspection and quality assessment using PyMOL software (version 3.1.6.1) to verify polypeptide chain integrity. Native ligands and non-essential heteroatoms were excised to furnish high-quality receptor structures for subsequent molecular docking investigations.

### 2.2. Deep Learning-Based Active Site Prediction

For systematic detection of prospective ligand-binding regions across protein topologies, we utilized UniSite, an advanced deep learning platform enabling automated cavity identification [25]. This algorithm, architected upon graph neural network frameworks, integrates geometric features and physicochemical properties of proteins for precise prediction. The operational workflow proceeded as follows: Initially, a PyTorch (Version 2.2.0)-based deep learning computational environment was established under the Linux operating system. Input target protein PDB files underwent standardized preprocessing, encompassing crystallographic water molecule removal, polar hydrogen atom addition, and energy minimization, subsequently transformed into spatial graph data structures encoding three-dimensional atomic coordinates, elemental identities, and chemical features. UniSite employs a physics-informed evolutionary operator that captures spatial proximity characteristics and residue charge distribution profiles, performing forward propagation via pretrained network weights to generate ligand-binding probability maps spanning complete protein surface meshes. Finally, spatial clustering analyses of high-probability scoring regions delineated discrete pocket coordinates. Among all identified candidate pockets, we comprehensively evaluated pocket volume, depth indices, and drug-likeness scores (Drug Score), selecting pockets with scores ≥0.9 and maximal volumes as target sites for subsequent virtual screening, typically corresponding to known ATP-binding domains or allosteric regulatory sites.

### 2.3. Assembly of Small-Molecule Natural Product Collections

Compound libraries for screening were sourced from the COCONUT natural products database (https://coconut.naturalproducts.net/), offering broad structural diversity among natural metabolites. A dataset containing roughly 695,000 molecular entries was acquired. To optimize screening productivity and enrichment rates, Lipinski’s Rule of Five (RO5) alongside QED scoring served as initial filters. Inclusion thresholds required: maximum one RO5 deviation and QED values exceeding 0.5 [26]. Furthermore, to investigate the distinctive chemical territory of marine-origin compounds, we obtained marine-specific subsets from CMNPD (https://cmnpd.org/) and implemented equivalent drug-likeness selection parameters.

### 2.4. Deep Learning-Driven High-Throughput Virtual Screening

This investigation employed the DiffDock deep learning docking platform for large-scale virtual screening. In contrast to conventional force field-based docking methodologies (e.g., AutoDock Vina, Glide), DiffDock harnesses diffusion model generative learning frameworks, exploring ligand binding poses and conformational spaces through reverse denoising processes, thereby achieving substantial improvements in both predictive accuracy and computational efficiency. For each protein-ligand pair, the DiffDock model generates multiple plausible binding poses, ranked according to confidence scores reflecting the model’s estimated pose accuracy. For each molecular entity, the highest-scoring (Rank 1) binding conformation was retrieved, with confidence scores serving as primary assessment criteria. Final candidate selection relied on comparative binding affinity evaluations to enable further in-depth characterization. It should be noted that DiffDock confidence scores are probabilistic estimates of pose prediction accuracy derived from the diffusion model’s reverse sampling process, and do not directly correlate with binding free energies or experimental affinity measurement. For binding affinity estimation.

### 2.5. Conventional Molecular Docking Validation and Interaction Profiling

To comprehensively elucidate interaction modalities between candidate compounds and target proteins, selected candidates underwent cross-validation via the conventional docking software QuickVina 2 (QVina2, version 2.1). QVina2, an enhanced iteration of AutoDock Vina, employs more efficient search algorithms while preserving the Vina scoring function’s accuracy. Post-docking, the optimal binding pose and corresponding binding energy (kcal/mol) were extracted for each compound, with more negative values indicating enhanced binding stability. By comparing DiffDock and QVina2 predictions, we assessed binding mode consistency and excluded compounds exhibiting unstable binding or significant discrepancies. Following initial analysis, PLIP (Protein-Ligand Interaction Profiler) facilitated generation of detailed interaction schematics capturing all non-covalent associations: hydrogen bonding, hydrophobic contacts, π-stacking arrangements, and ionic bridges. Subsequently, pharmacophoric elements were manually delineated and rendered in PyMOL according to interaction patterns, clarifying functional contributions of essential structural fragments.

### 2.6. Assembly of Molecular Dynamics Simulation Frameworks

Simulation systems originated from top-ranked binding geometries generated by DiffDock. Protein parameterization employed the AMBER99SB-ILDN force field framework. Ligand topological parameters were constructed using Sobtop (version 1.0 dev3.2). Atomic partial charges for small molecules were determined through RESP (restrained electrostatic potential) fitting implemented in Multiwfn [27,28]. The procedure comprised: initial geometry optimization and single-point energy evaluation at the B3LYP/6-31G* level via ORCA quantum chemistry software (version 5.0.4), producing electrostatic potential grids; Multiwfn then processed these outputs to derive RESP charges; these fitted charges were subsequently incorporated into Sobtop-generated topology descriptors. Protein-ligand assemblies were positioned centrally within cubic simulation cells maintaining ≥10 Å separation between solute surfaces and cell boundaries, then solvated using TIP3P water molecules. System electroneutrality was achieved through addition of appropriate Na^+^ or Cl^−^ counterions.

### 2.7. Energy Minimization and Equilibration Protocols

Initial energy minimization eliminated sterically unfavorable atomic proximities via steepest descent optimization, converging at maximum forces below 1000 kJ/(mol·nm). Subsequent thermal ramping elevated system temperature from 0 to 300 K across 100 ps under NVT (constant volume-temperature) conditions, applying 1000 kJ/(mol·nm^2^) positional restraints to protein heavy atoms. An additional 100 ps equilibration phase under NPT (constant pressure-temperature) ensemble followed, utilizing Berendsen or Parrinello-Rahman barostats to stabilize pressure at 1 bar while maintaining 300 K, enabling density relaxation.

### 2.8. Production-Phase Molecular Dynamics Trajectories

Dynamic stability assessment and binding mode characterization of protein-ligand assemblies involved 200 ns unrestrained MD simulations executed via GROMACS (version 2024.4). Integration proceeded with 2 fs timesteps, employing LINCS constraints for all covalent bonds. Thermal and pressure regulation utilized v-rescale thermostat and Parrinello-Rahman barostat coupling schemes, respectively. Short-range van der Waals forces applied a 10 Å truncation distance, whereas long-range electrostatics were handled through Particle Mesh Ewald (PME) summation. Coordinate snapshots were archived at 10 ps intervals for downstream analysis.

### 2.9. Binding Affinity Estimation Protocols

Quantitative affinity assessment between candidate ligands and target proteins utilized MM-GBSA (Molecular Mechanics/Generalized Born Surface Area) calculations. This endpoint methodology efficiently estimates binding strengths by energetically evaluating MD trajectory snapshots, balancing computational efficiency with predictive accuracy. The gmx trjconv utility extracted 100 terminal frames from 200 ns production runs for analysis. Free energy computations proceeded via gmx_MMPBSA, wherein gas-phase interaction energies (including molecular mechanics and van der Waals components) were evaluated through the sander module employing AMBER99SB-ILDN parameters. Solvation contributions utilized the GB-OBC1 implicit solvent model (igb = 5), while nonpolar solvation terms derived from SASA (solvent-accessible surface area) calculations. It should be noted that the reported MM-GBSA values represent binding enthalpies (ΔH), as configurational entropy corrections (via normal mode analysis or quasi-harmonic methods) were not performed. Entropy calculations are computationally demanding and introduce substantial uncertainty; therefore, for comparative analysis purposes, we report enthalpic components only. Absolute values should be interpreted cautiously, though relative comparisons between targets remain valid under the assumption of similar entropy contributions.

### 2.10. Quantum Chemical Analysis via Density Functional Theory

Electronic-level characterization of candidate molecular properties and reactivity employed DFT (density functional theory) approaches. Quantum chemical evaluations utilized the ORCA 5.0.4 computational suite. Initial gas-phase geometry optimizations proceeded at B3LYP/def2-TZVP theoretical rigor, with subsequent harmonic frequency analysis confirming true minima (no imaginary modes). Optimized structures enabled electronic structure determination, emphasizing frontier molecular orbital analysis: HOMO (highest occupied molecular orbital) and LUMO (lowest unoccupied molecular orbital). HOMO characterizes electron donation propensity, LUMO reflects electron acceptance capacity, while their energetic separation (HOMO-LUMO gap, ΔE) critically indicates chemical stability and reactivity potential. Narrower gaps correlate with enhanced polarizability and facilitated electronic transitions, though potentially compromising kinetic persistence. Visualization of computational outputs, encompassing MESP (molecular electrostatic potential) surfaces and frontier orbital distributions, was accomplished using Multiwfn 3.8_dev.

## 3. Results

### 3.1. Active Site Identification

**Figure 1:**
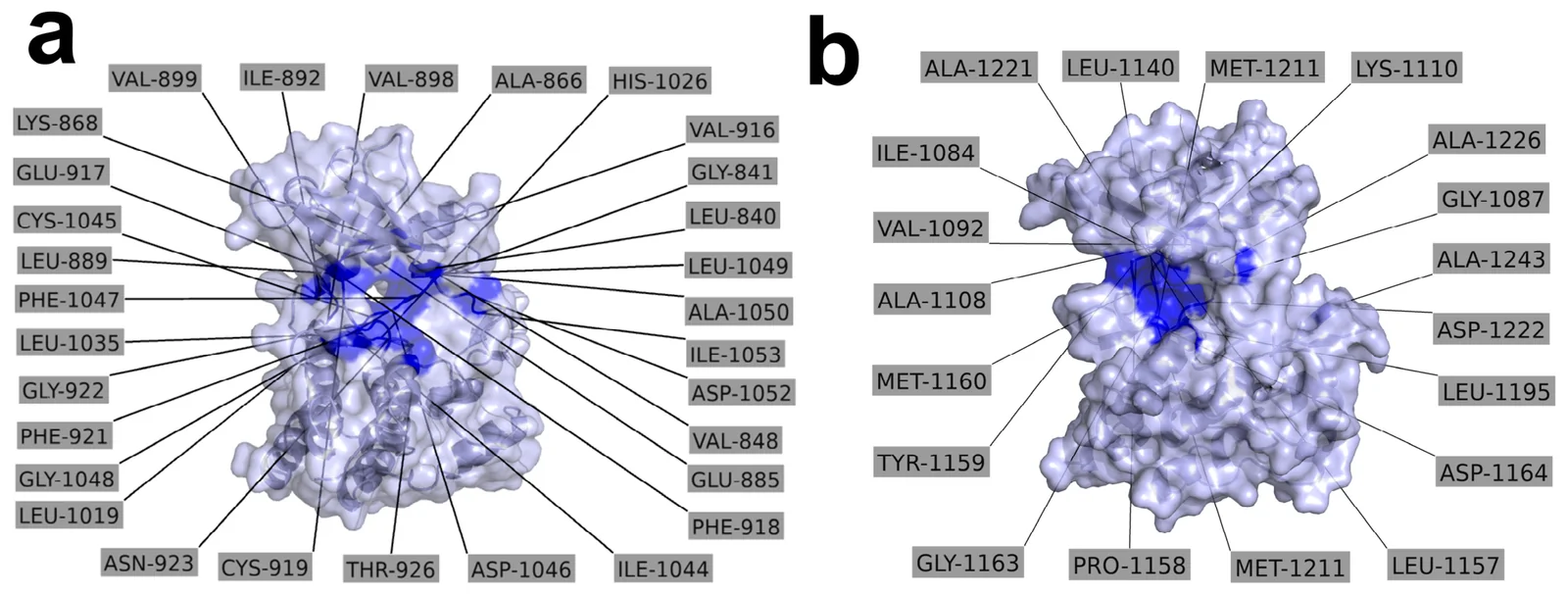
Ligand-binding cavities of VEGFR2 and c-Met identified through UniSite prediction. (**a**) High-confidence binding sites (drug scores ≥ 0.9) visualized on VEGFR2 protein topology. (**b**) High-confidence binding sites (drug scores ≥ 0.9) visualized on c-Met protein topology. Regions colored in deep blue indicate anticipated binding locations within domain interfaces, characterized by balanced hydrophobic and polar surface properties. Key residues forming the binding pockets are labeled.

The UniSite model identified 14 potential binding pockets for both VEGFR2 and c-Met (Table S1). These pockets exhibited variations in spatial distribution across protein surfaces, volumetric dimensions, and drug-likeness scoring. To ensure accuracy and biological relevance of subsequent virtual screening, we selected high-confidence pockets with Drug Scores ≥ 0.9 as target sites. This scoring threshold guaranteed that selected pockets possessed suitable geometric and chemical environments for small molecule drug binding. Visualization of one potential active pocket for VEGFR2 (Figure 1a) and one for c-Met (Figure 1b) revealed deep blue regions representing potential active sites. Such binding sites resided within pronounced interdomain crevices, displaying balanced distributions of hydrophobic and polar surfaces characteristic of typical small-molecule accommodation cavities.

### 3.2. Virtual Compound Library Construction

Drug-likeness evaluation was performed on the primary natural product compound collection. Applying Lipinski’s Rule of Five criteria alongside QED scoring metrics, preliminary virtual screening encompassed 700,000 molecular entities, yielding 37,280 qualifying natural products and an additional 47,450 candidates from marine-derived subsets. Subsequently, using the cheminformatics tool RDKit (version Q3 2025), one-dimensional SMILES representations of these 84,730 total small molecule compounds were converted to three-dimensional structures. AutoDock Tools software (version 1.5.7), Tools software was employed to preprocess all 3D small molecule structures, including polar hydrogen addition and charge calculation, ultimately saving them in the PDBQT format required for docking.

### 3.3. AI-Assisted Drug Screening

Leveraging DiffDock, we carried out a deep learning-driven protein-ligand binding assessment involving the target proteins and 37,280 ligands from the curated COCONUT database, alongside 47,450 ligands from CMNPD. Binding poses were evaluated using DiffDock confidence scores, which reflect the model’s estimated accuracy of pose prediction rather than binding affinity. Following the recommendations from the DiffDock developers, we used confidence score thresholds for pose quality filtering: values below −1.5 indicate low-confidence poses likely to be inaccurate, scores between −1.5 and 0 suggest moderate prediction confidence, while values exceeding 0 represent high-confidence pose predictions. It is important to emphasize that these thresholds assess pose reliability, not interaction strength. To pinpoint small-molecule drugs exhibiting the highest dual affinity for both target proteins, we performed an intersection analysis of prospective candidates against VEGFR2 and c-Met. This approach yielded 6870 compounds with confidence scores exceeding −1.5 from COCONUT (Table S2) and 3900 compounds meeting identical criteria from CMNPD (Table S3). Building on this foundation, we further refined our selection by applying a threshold whereby the mean confidence score across both target proteins exceeded 0. Through this stringent filtering process, we ultimately uncovered 2 compounds from the natural product repository and an additional 2 from the marine-derived natural product collection—each demonstrating the capacity to concurrently engage both VEGFR2 and c-Met, culminating in a total of 4 promising small-molecule candidates (Table 1).

InChIKey, IUPAC International Chemical Identifier hash key serves as a unique, database-searchable molecular fingerprint for unambiguous compound identification and cross-database deduplication; MW, Molecular weight (Da); ALogP, Calculated octanol–water partition coefficient; RB, Number of rotatable bonds; HBA, Number of hydrogen bond acceptors; HBD, Number of hydrogen bond donors; TPSA, Topological polar surface area (Å^2^); Ar. Rings, Number of aromatic rings; HA, Number of heavy (non-hydrogen) atoms; QED, Quantitative estimate of drug-likeness. ALogP was computed using the Ghose–Crippen–Viswanadhan atom-type approach. Compounds satisfying Lipinski’s rule of five (MW ≤ 500 Da, ALogP ≤ 5, HBA ≤ 10, HBD ≤ 5) and Veber’s criteria (RB ≤ 10, TPSA ≤ 140 Å^2^) are considered to possess favorable oral bioavailability profiles. QED scores were calculated according to the weighted scheme proposed by Bickerton et al. Which integrates eight molecular descriptors—MW, ALogP, HBA, HBD, TPSA, RB, aromatic ring count, and the number of structural alerts—into a single composite metric reflecting overall drug-likeness. A QED value closer to 1 indicates a higher resemblance to approved oral drugs.

These molecules were subjected to molecular docking using QVina2 [29] and binding energy (BE) calculations (Table 2). A negative BE value indicates a favorable protein-ligand interaction; the larger the negative value, the stronger the binding affinity. Conversely, values close to or above zero indicate a weaker or unfavorable binding. Following rigorous screening, we found that CNP0376925.2 and CNP0073361.1 failed to form stable binding with receptors, ultimately identifying CMNPD30506 as the core candidate drug exhibiting favorable predicted binding affinity with both VEGFR2 and c-Met target proteins.

### 3.4. Binding Mode Analysis

**Figure 2:**
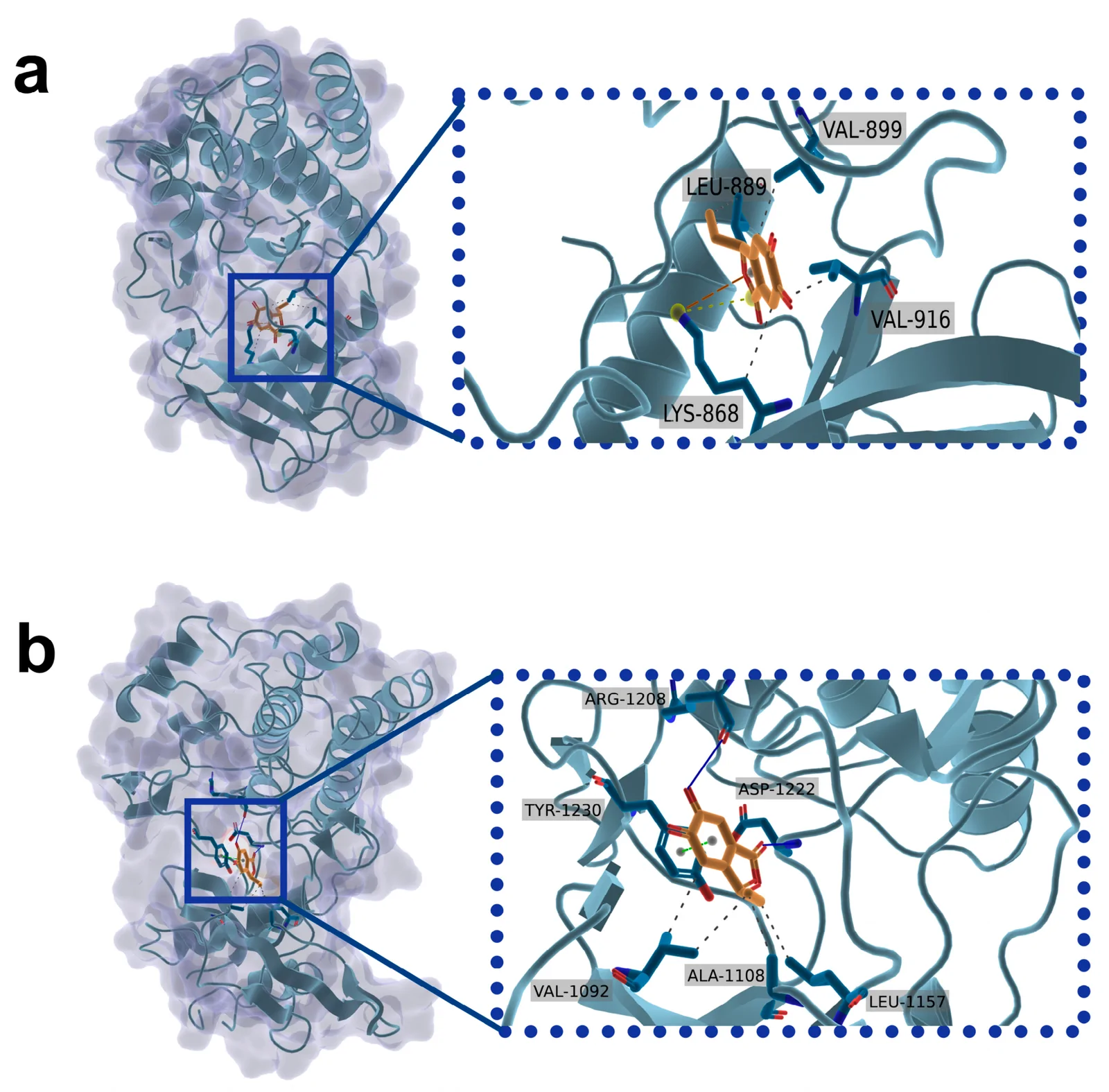
Non-covalent interaction profiles of CMNPD30506 with VEGFR2 and c-Met. Molecular interactions analyzed by PLIP and visualized in PyMOL (v3.1.6.1). Ligands and key residues shown as stick models; hydrogen bonds (blue lines), hydrophobic interactions (gray dashed lines), π-π stacking (green dashed lines), π-cation interactions (orange dashed lines), and salt bridges (yellow dashed lines). Electrostatic potential surfaces illustrate binding contexts. (**a**) CMNPD30506–VEGFR2 complex. (**b**) CMNPD30506–c-Met complex.

To precisely elucidate binding modes and pharmacophoric features of core ligands with target proteins, we employed the Protein-Ligand Interaction Profiler (PLIP) tool for comprehensive non-covalent interaction assessment of optimal docked complex conformations. Through PLIP algorithms, hydrogen bonding networks, hydrophobic interactions, electrostatic interactions, and π-π stacking effects between ligands and receptor binding pockets were systematically extracted and quantified. In mode analyses, ligands and key interacting residues were highlighted as stick models, intuitively presenting spatial positions of hydrogen bonds and π-π stacking. Additionally, electrostatic potential surfaces of receptor proteins were constructed to demonstrate docking relationships. Analyses revealed that CMNPD30506 forms 4 hydrophobic interactions with key residues LEU889, VAL899, and VAL916 of VEGFR2, along with one π-cation interaction between the aromatic benzene ring and the positively charged ε-amino group of LYS868. PLIP initially flagged an additional electrostatic interaction involving LYS868; however, upon chemical structure review, CMNPD30506 lacks ionizable groups that would form a classical salt bridge under physiological pH. This interaction more likely represents a polar contact between the phenolic hydroxyl group and the lysine side chain, or a water-mediated hydrogen bonding network. (Figure 2a); with c-Met ‘s 6 key amino acids (TYR1230, ARG1208, ASP1222, ILE1157, ALA1108, and VAL1092), it establishes 4 hydrophobic interactions, 2 hydrogen bonds, and 1 π-π stacking interaction (Figure 2b). These findings indicate that CMNPD30506 can form relatively stable docking with both target proteins.

### 3.5. Molecular Dynamics Simulations

**Figure 3:**
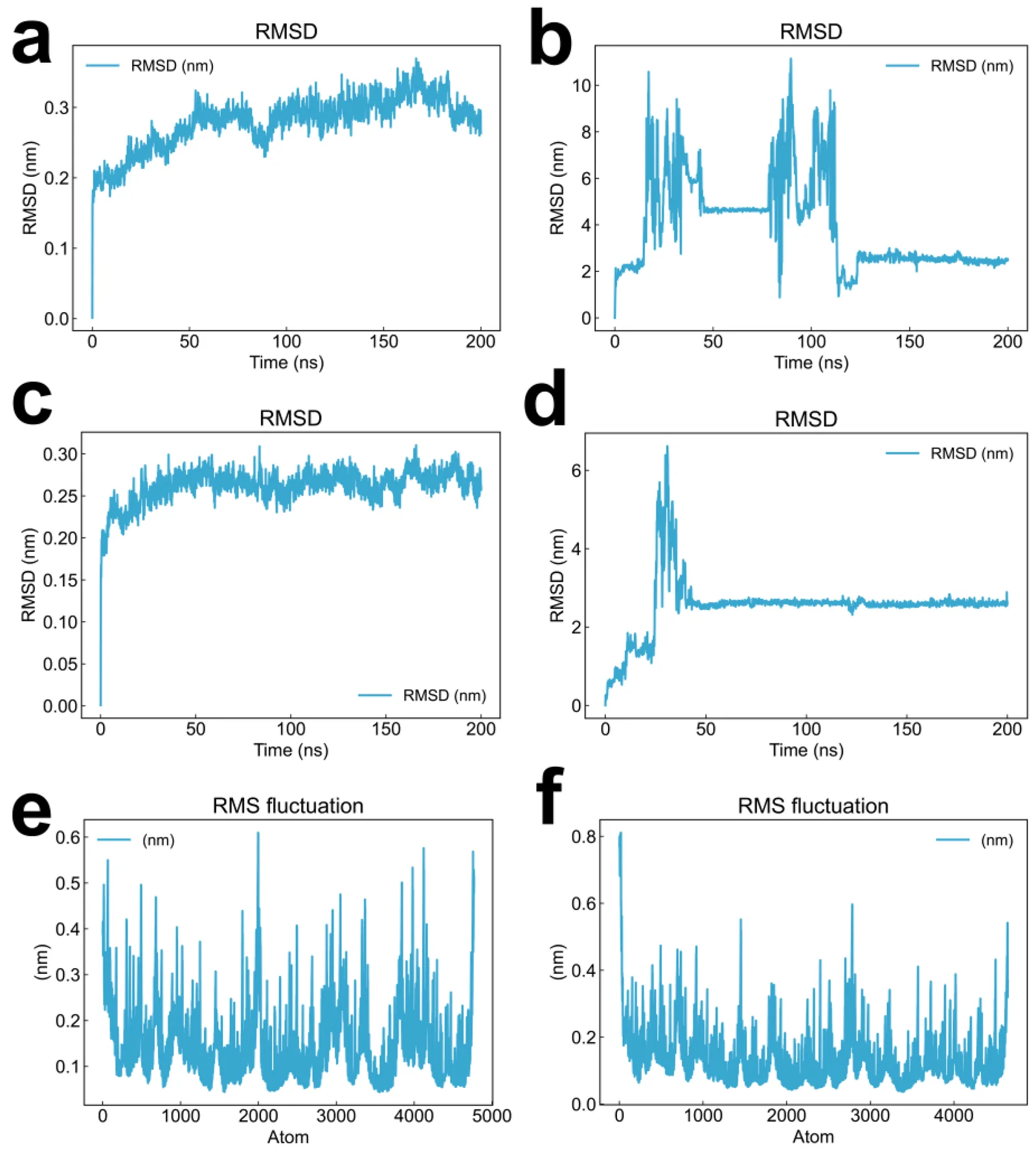
Structural stability metrics from 200 ns molecular dynamics simulations. (**a**–**d**) Root-mean-square deviation (RMSD) of protein backbone and ligand atoms over time for: (**a**) VEGFR2 backbone, (**b**) ligand in VEGFR2 complex, (**c**) c-Met backbone, and (**d**) ligand in c-Met complex. (**e**,**f**) Root-mean-square fluctuation (RMSF) per residue for: (**e**) VEGFR2–CMNPD30506 and (**f**) c-Met–CMNPD30506 complexes.

Based on the screening workflow described above, we computationally predicted CMNPD30506 as a potential dual VEGFR2 and c-Met inhibitor candidate. Subsequently, we conducted 200 ns molecular dynamics simulations to validate binding stability. Examination of root mean square deviation (RMSD) curves revealed that in the VEGFR2-CMNPD30506 system, both protein backbone (Figure 3a) and ligand (Figure 3b) RMSD curves exhibited pronounced stability in the latter simulation phase. Similarly, in the c-Met -CMNPD30506 system, protein backbone (Figure 3c) and ligand (Figure 3d) RMSD curves demonstrated comparable stability. The ligand RMSD trajectories (Figure 3b,d) warrant careful interpretation. Both systems exhibit pronounced RMSD fluctuations during the initial simulation phase. These early-phase fluctuations should not be interpreted as binding instability or ligand dissociation. Rather, they reflect: ligand conformational adaptation from the rigid docking pose to the dynamic solution environment, binding mode refinement as the ligand optimizes interactions within the ATP pocket, and reference-frame effects where ligand rotation/translation within the pocket can inflate RMSD values calculated after protein backbone alignment.

Root mean square fluctuation (RMSF) reflects positional fluctuation magnitudes of individual protein residues during simulations, with elevated RMSF values typically corresponding to flexible loop regions or solvent-exposed loops, while diminished values correlate with domain cores or secondary structural elements. RMSF analyses demonstrated that in both VEGFR2 (Figure 3e) and c-Met (Figure 3f) binding systems with CMNPD30506, residue and small molecule fluctuations remained minimal, signifying maintenance of relatively high structural stability throughout simulations.

**Figure 4:**
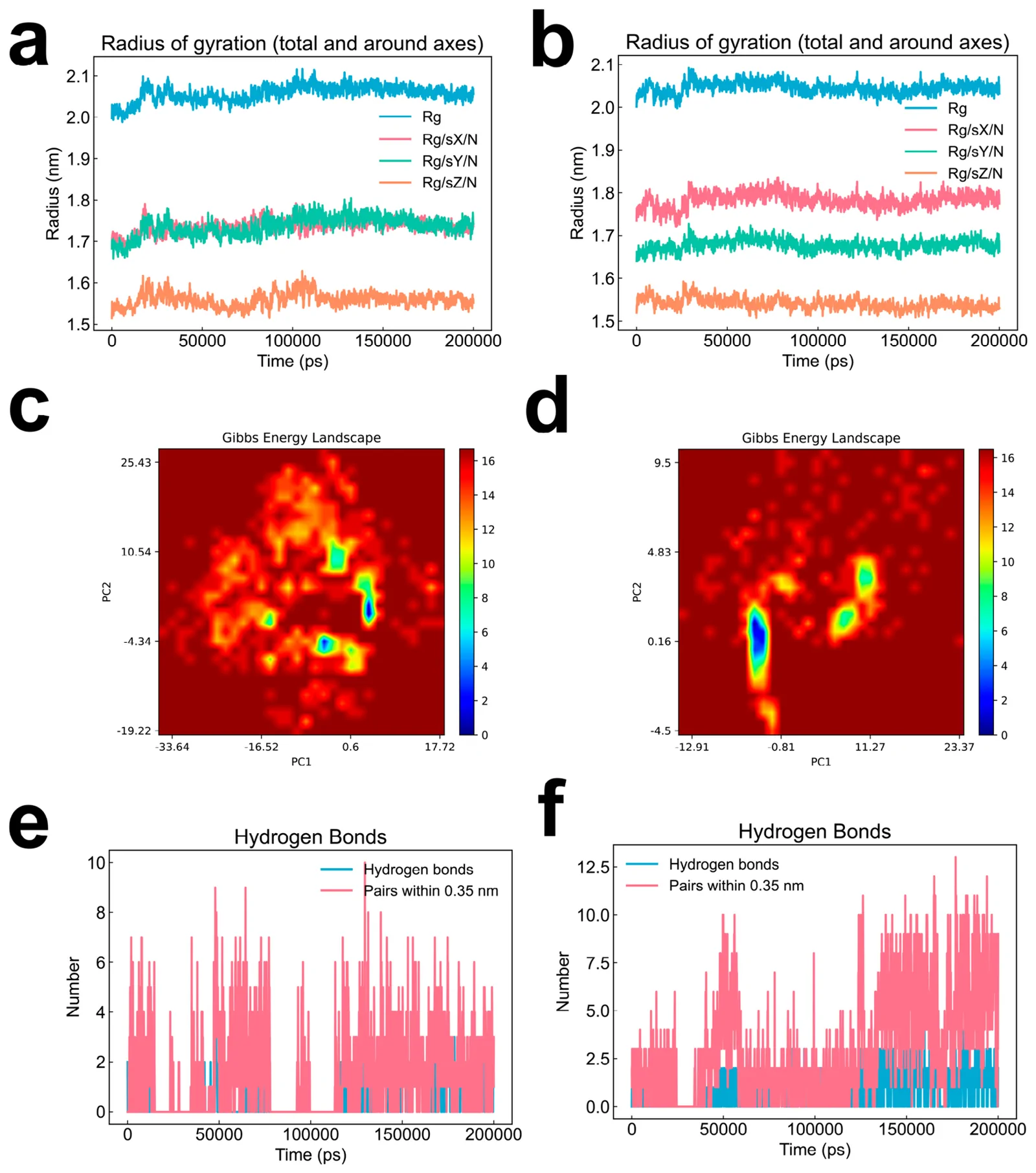
Radius of gyration, free energy landscapes, and hydrogen bonding dynamics. (**a**,**b**) Radius of gyration (Rg) over simulation time for: (**a**) VEGFR2–CMNPD30506 and (**b**) c-Met–CMNPD30506 complexes, indicating maintained protein compactness. (**c**,**d**) Gibbs free energy landscapes projected onto principal components PC1 and PC2 for: (**c**) VEGFR2–CMNPD30506 and (**d**) c-Met–CMNPD30506 complexes. Blue regions represent dominant low-energy conformational states. (**e**,**f**) Time evolution of intermolecular hydrogen bonds for: (**e**) VEGFR2–CMNPD30506 and (**f**) c-Met–CMNPD30506 systems.

Radius of gyration (Rg) quantifies protein mass distribution dispersal relative to the center of mass, serving as a sensitive indicator of overall protein compactness and folding state. For well-folded globular proteins, Rg values should stabilize within ranges commensurate with molecular weight; abrupt Rg increases typically signify protein unfolding or domain dissociation. Following small molecule binding, both VEGFR2 (Figure 4a) and c-Met (Figure 4b) exhibited minimal Rg fluctuations, confirming maintenance of compact folded states and indicating robust structural compactness and stability.

Two-dimensional Gibbs free energy landscapes constructed via principal component analysis (PCA) provide panoramic views of energy distributions under major conformational motion modes. These analyses project high-dimensional conformational spaces onto two-dimensional planes (Figure 4c,d), displaying energy distribution landscapes under different principal components, where deeper blue coloration indicates ligand-protein complexes reaching energy minima and enhanced binding stability. Both systems exhibited single or adjacent blue energy basins, lacking secondary energy minima or multiple basins, indicating that complexes predominantly occupied dominant conformational states during simulations without substantial conformational transitions or polymorphism.

Hydrogen bond analyses revealed that both VEGFR2 (Figure 4e) and c-Met (Figure 4f) systems with CMNPD30506 generated hydrogen bonds during simulations. While the VEGFR2-CMNPD30506 system (Figure 4e) initially exhibited fewer hydrogen bonds—seemingly contradicting PLIP static analyses that failed to identify prominent hydrogen bonds—MD analyses unveiled dynamic realities: initial docking poses may have lacked stable hydrogen bonds, yet under solution environments, protein side-chain rearrangements and ligand adjustments enabled intermittent weak hydrogen bond formation. During the latter simulation phase (100–200 ns), hydrogen bond numbers slightly increased, reflecting the system’s tendency toward more favorable interaction modes. The c-Met-CMNPD30506 system (Figure 4f) demonstrated more pronounced hydrogen bond enrichment characteristics. Around 50 ns of simulation, hydrogen bond numbers stabilized at approximately 2, consistent with 1–2 hydrogen bonds identified by static analyses. Remarkably, during the latter simulation phase (120–200 ns), hydrogen bond numbers substantially increased and stabilized at 2–3. This hydrogen bond network enhancement phenomenon may originate from the following mechanisms: (1) initially unrecognized potential hydrogen bond donors/acceptors adjusting positions under dynamic environments to form new hydrogen bonds; (2) structural water molecule-mediated protein-ligand indirect hydrogen bonding bridges; (3) ligand conformational fine-tuning enabling polar groups to more precisely align with receptor hydrogen bond sites. Hydrogen bond analyses revealed differential binding strategies of the ligand toward both targets, suggesting predominant reliance on hydrophobic and π-cation interactions in VEGFR2 while forming more extensive hydrogen bonding networks in c-Met. These patterns—combined with early-phase ligand RMSD fluctuations suggest that the ligand underwent conformational rearrangement within the binding pocket during the simulation. This behavior is consistent with induced-fit binding mechanisms commonly observed in kinase-inhibitor systems. The initial docking poses, generated by static energy minimization, may not have captured the thermodynamically optimal binding mode achievable under dynamic solution conditions. During MD simulations, the ligand appears to have explored conformational space within the binding pocket, adjusting its position and orientation to optimize interactions, ultimately settling into alternative binding modes characterized by enhanced hydrogen bonding networks and reduced RMSD fluctuations.

### 3.6. Binding Free Energy Calculations

**Figure 5:**
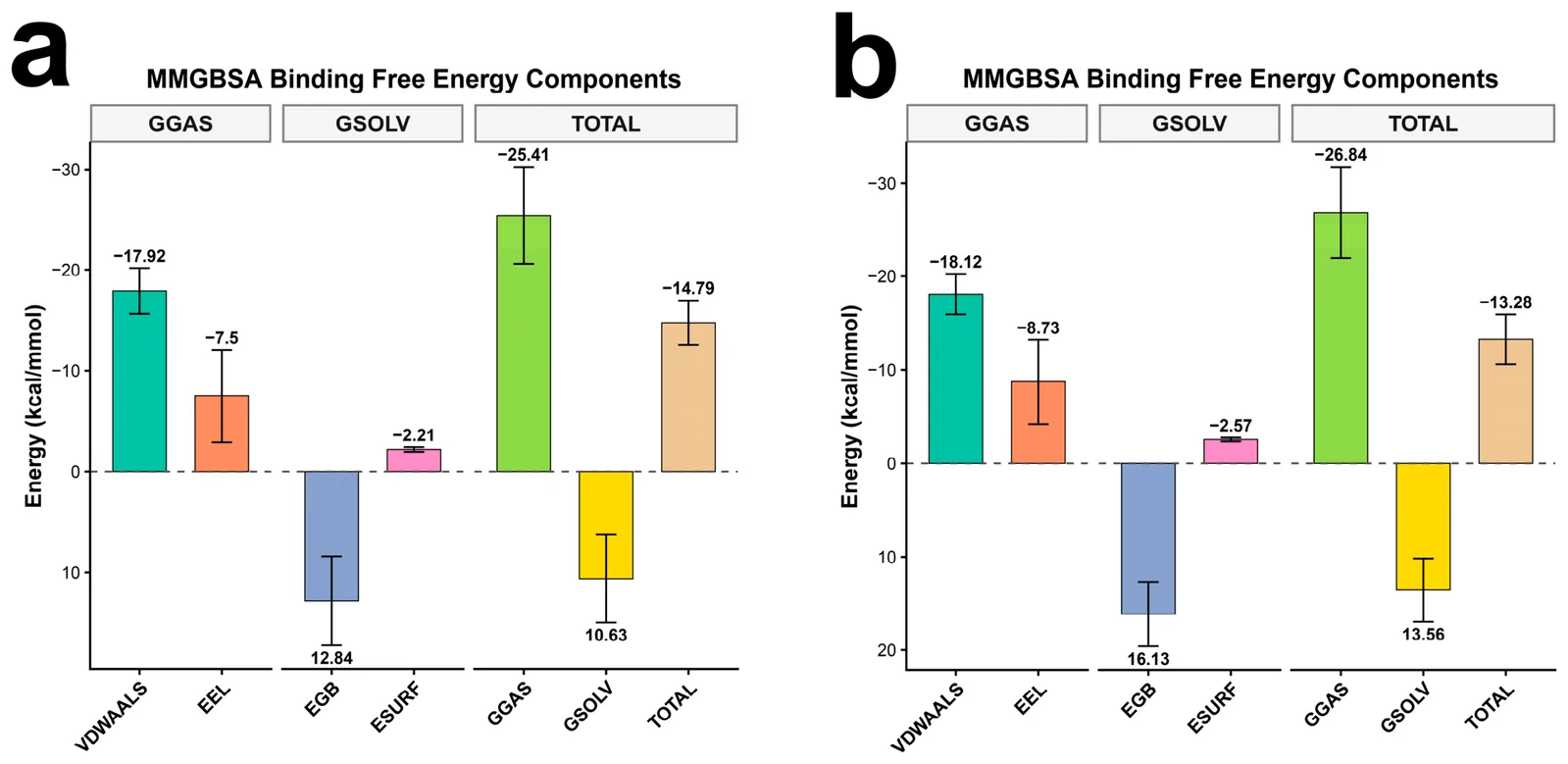
Energetic decomposition of MM-GBSA-calculated binding affinities for dual-target systems. Component analysis displaying van der Waals (ΔVDWAALS), electrostatic (ΔEEL), polar solvation (ΔEGB), and nonpolar solvation (ΔESURF) contributions in: (**a**) VEGFR2–CMNPD30506 interaction (ΔG_bind = −14.79 kcal/mol) and (**b**) c-Met–CMNPD30506 interaction (ΔG_bind = −13.28 kcal/mol). The total binding free energy is computed as: GGAS + GSOLV, where GGAS = ΔVDWAALS + ΔEEL represents gas-phase interaction energy, and GSOLV = ΔEGB + ΔESURF represents solvation free energy. Standard deviation (SD) indicated by error bars.

To systematically evaluate binding affinities of candidate compounds with dual targets, we calculated binding free energies of CMNPD30506 with VEGFR2 and c-Met using the MM-GBSA methodology, accompanied by energy decomposition analyses (Figure 5a,b). Energy decomposition results for both complexes consistently demonstrated that van der Waals interactions (ΔVDWAALS) constitute the primary thermodynamic driving forces for ligand-protein binding, yielding values of −17.92 and −18.12 kcal/mol, respectively, reflecting shape complementarity between candidate molecules and binding pockets of both targets. Although electrostatic interactions (ΔEEL) provided favorable contributions in the gas phase (−7.5 and −8.73 kcal/mol), they were substantially offset by polar desolvation penalties (ΔEGB = +12.84 and +16.13 kcal/mol), with net polar contributions proving unfavorable for binding across all systems—a phenomenon consistent with thermodynamic characteristics of most protein-ligand interaction systems. Non-polar solvation terms (ΔESURF = −2.21 and −2.57 kcal/mol) furnished moderate yet consistent favorable contributions through hydrophobic effects across all systems.

From a dual-target comparative perspective, CMNPD30506 exhibited total binding free energies of −14.79 and −13.28 kcal/mol for VEGFR2 and c-Met, respectively. These computational estimates suggest thermodynamically stable binding modes for both complexes under the simulation conditions employed. Both values are negative, indicating thermodynamically favorable computational binding estimates. However, as MM-GBSA values are relative metrics dependent on force field parameters and simulation protocols, these values cannot be directly compared to experimental binding affinities. The observed 1.51 kcal/mol differential in predicted binding free energy between VEGFR2 and c-Met suggests comparable computational affinity for both targets. However, as selectivity against other kinase family members has not been assessed, we cannot conclude whether this represents a favorable or unfavorable selectivity profile from a therapeutic perspective.

Collectively, both complexes exhibited favorable thermodynamic stability, further substantiating the feasibility of CMNPD30506 as a dual VEGFR2/ c-Met lead compound.

### 3.7. Density Functional Theory (DFT) Calculations of Small Molecule Drugs

**Figure 6:**
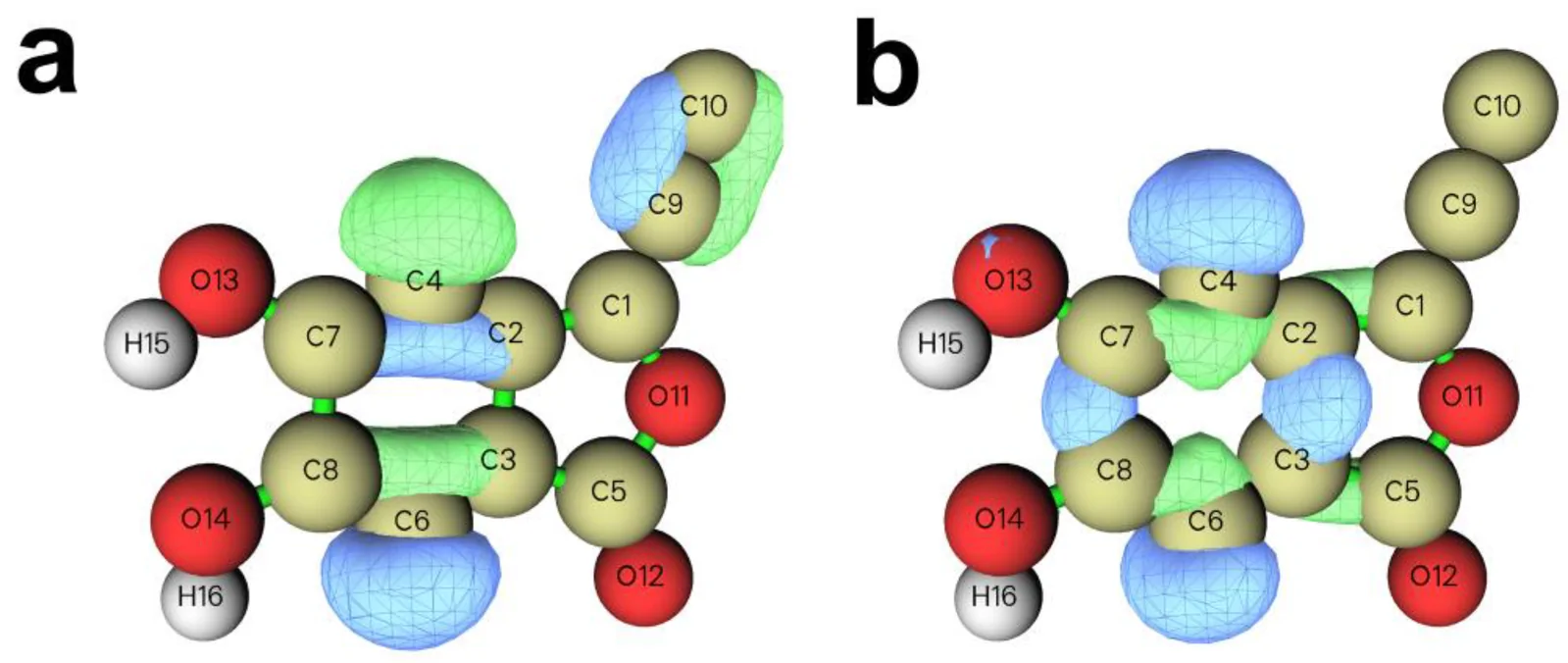
Frontier molecular orbital distributions of CMNPD30506 from DFT calculations. Isosurface plots (isovalue = 0.02 a.u.) displaying spatial distributions of: (**a**) LUMO (−5.076 eV) localized to benzene ring and ethyl group, and (**b**) HOMO (−7.486 eV) localized to benzene ring π-system. HOMO-LUMO energy gap: 2.410 eV.

To comprehensively dissect chemical reactivity and drug development potential of candidate compounds at the electronic structure level, we conducted density functional theory (DFT) calculations on CMNPD30506, emphasizing analyses of frontier molecular orbital (FMO) energies and spatial distributions. Frontier molecular orbital analysis of CMNPD30506 revealed a HOMO energy of −7.486 eV, LUMO energy of −5.076 eV, and HOMO-LUMO energy gap (ΔE) of 2.410 eV. This moderately low energy gap indicates favorable electronic responsiveness and appropriate polarization characteristics, facilitating electronic redistribution during target protein binding processes to enhance interaction stability. Regarding orbital spatial distributions, the LUMO predominantly localizes to benzene ring and ethyl group (Figure 6a), suggesting these domains may function as electron acceptors participating in stabilizing interactions during binding while potentially augmenting molecular lipophilicity and steric shielding effects. The HOMO primarily localizes to benzene ring regions (Figure 6b), indicating this π-electron-enriched domain serves as the principal molecular electron donor site, capable of engaging positively charged or electron-deficient amino acid residues in VEGFR2 or c-Met active pockets through π-π stacking or cation-π interactions, concordant with molecular docking results. However, the relatively small ΔE value, while advantageous for enhancing molecular electronic adaptability, also suggests potential oxidative metabolic susceptibility in vivo.

## 4. Discussion

Phthalide natural products have been documented to exhibit diverse biological activities, including neuroprotection [30] and antitumor effects [31,32]. Particularly noteworthy, several phthalide derivatives have advanced into clinical application or investigational stages. For instance, 3-n-butylphthalide (NBP) is approved for ischemic stroke treatment, ameliorating cerebral blood flow and neurological function through multi-target mechanisms [33]. Ligustilide and related phthalide compounds demonstrate tumor cell proliferation inhibitory activities through modulation of PI3K/AKT and MAPK signaling pathways [34].

Notably, CMNPD30506 has been previously reported to exhibit moderate cytotoxic activity against renal cancer cell lines ACHN and 786-O [35], while CMNPD30538 showed weak antiproliferative activity in prior phenotypic screens [36]. However, these studies did not specifically evaluate kinase inhibitory activity or assess effects on c-Met/VEGFR2 signaling pathways. The micromolar cytotoxicity observed for CMNPD30506 falls within acceptable ranges for hit compounds amenable to structure-guided optimization, and experimental validation through biochemical kinase assays remains essential to confirm whether our computational binding predictions translate to functional target inhibition.

This discovery not only furnishes novel candidate molecules for dual-target HCC therapy but also exemplifies the application value of AI-driven drug discovery paradigms in multi-target pharmaceutical development.

Our molecular docking and interaction analyses unveiled distinctive binding architectures of CMNPD30506 with both target kinase domains. Intriguingly, CMNPD30506 manifests more diverse interaction modalities within the c-Met binding pocket, encompassing π-π stacking with TYR1230, hydrogen bonding with ARG1208 and ASP1222, alongside multiple hydrophobic contacts. TYR1230 represents a pivotal residue within the c-Met kinase domain, with its phosphorylation status directly influencing kinase activity. The candidate compound’s engagement with this residue via π-π stacking may exert stabilizing effects on c-Met’s active conformation. Furthermore, hydrogen bonding with ASP1222 suggests CMNPD30506 may exhibit type II inhibitor characteristics, capable of stabilizing inactive kinase conformations [37,38]. Type II inhibitors typically demonstrate enhanced selectivity and more sustained inhibitory effects [39].

The pronounced ligand RMSD fluctuations observed during the initial simulation phases merit careful interpretation in the context of kinase-inhibitor binding dynamics. Rather than indicating binding instability, these fluctuations are characteristic of induced-fit binding processes where the ligand undergoes conformational rearrangement to optimize interactions with a flexible binding pocket. RMSF analyses further substantiated low fluctuation magnitudes of binding pocket residues, indicating ligand binding confers local structural stabilization. Rg value stability reflects preserved protein overall folding integrity, critical for maintaining functional kinase domain conformations.

Hydrogen bond analyses warrant particular attention. While initial docking revealed fewer hydrogen bonds between CMNPD30506 and VEGFR2, MD simulations demonstrated sustained hydrogen bonding networks, indicating hydrophobic interactions and π-cation interactions play dominant roles in maintaining complex stability. Conversely, the c-Met-CMNPD30506 system manifested increased hydrogen bond numbers during latter simulation phases, potentially attributable to dynamic protein conformational adjustments enabling additional polar residues to engage in ligand interactions. This induced-fit mechanism has been observed in binding processes of numerous kinase inhibitors [40]. Increased hydrogen bonding also resonates with the larger electrostatic contributions exhibited by this system in binding free energy calculations.

PCA-based free energy landscapes furnished holistic views of complex energy distributions across conformational space. Both systems exhibited distinct energy minimum regions (deep blue), hallmark signatures of stable binding. Unlike multi-peak energy distributions characteristic of unstable complexes, our results indicate ligands do not undergo significant binding mode transitions within binding pockets, further supporting binding stability.

MM-GBSA-calculated binding free energies (−14.79 and −13.28 kcal/mol) align with theoretical value ranges for inhibitors [41,42]. Energy decomposition analyses elucidated key thermodynamic factors driving binding. Van der Waals interactions (approximately −18 kcal/mol) constituted the most substantial favorable contribution term across both systems, consonant with extensive hydrophobic contacts observed in docking analyses. RTK inhibitors typically possess hydrophobic chemical structures to achieve optimal matching with ATP pocket hydrophobic regions [43].

Electrostatic interactions furnished moderate favorable contributions in the gas phase yet were substantially offset by polar desolvation penalties during solvation—a commonplace phenomenon in protein-ligand binding. The unfavorable nature of polar contributions suggests that while hydrogen bonds and polar interactions prove critical for binding specificity, they do not constitute primary driving forces. Non-polar solvation terms provided approximately −2.5 kcal/mol stabilization contributions through hydrophobic effects, reflecting reduced solvent-accessible surface area upon ligand binding.

From a dual-target comparative perspective, CMNPD30506 exhibited marginally superior binding free energy toward VEGFR2 compared to c-Met (differential of approximately 1.5 kcal/mol), yet both fell within binding ranges. This balanced dual-target affinity represents ideal multi-target inhibitor characteristics [44], ensuring simultaneous effective inhibition of both pathways while circumventing selectivity window issues potentially arising from excessively robust binding to either target. In contrast, some multi-target kinase inhibitors exhibit substantial affinity differentials across distinct targets, rendering genuine multi-target blockade challenging in clinical applications.

DFT-revealed frontier molecular orbital characteristics furnished crucial insights into CMNPD30506’s electronic structure and reactivity. The HOMO-LUMO energy gap of 2.410 eV occupies a moderate range, indicating the molecule neither exhibits excessive reactivity due to excessively small gaps nor lacks electronic responsiveness from excessively large gaps [45]. This moderate energy gap facilitates appropriate electronic rearrangements during protein binding, thereby enhancing interactions.

Observations of HOMO predominant distribution in benzene ring regions align with this domain’s function as an electron donor participating in π-π stacking and cation-π interactions. In c-Met binding, HOMO-enriched regions engage TYR1230 in π-π stacking; in VEGFR2 binding, they interact with LYS868 through π-cation mechanisms—both dependent upon aromatic ring electron cloud distributions. LUMO distribution in acceptor sites suggests these regions may interact with electron-rich residues or polarized water molecules in protein pockets.

Nevertheless, DFT calculations also unveiled potential drug development challenges. Relatively small energy gaps may render molecules susceptible to oxidative metabolic enzymes—a common issue in natural product-derived pharmaceuticals. Future chemical optimizations could consider introducing electron-modulating groups to moderately increase energy gaps while preserving key pharmacophores, thereby improving metabolic stability. An important limitation of our DFT analysis is that all quantum chemical calculations were performed under gas-phase conditions without accounting for solvation effects. In reality, drug molecules experience aqueous solvation in biological fluids and desolvation upon binding to hydrophobic protein pockets. Implicit solvation models can capture bulk solvent effects on frontier molecular orbital energies and gap values. Polar solvents typically reduce HOMO-LUMO gaps through differential stabilization of occupied and virtual orbitals, potentially affecting our interpretation of electronic reactivity and metabolic susceptibility. Furthermore, orbital localization patterns observed in gas phase may shift in response to solvent polarity. Future computational studies should incorporate implicit or explicit solvation models in DFT calculations to provide more realistic estimates of electronic properties under physiologically relevant conditions.

CMNPD30506’s derivation from marine natural product databases underscores the significance of marine biological resources in pharmaceutical development. The high salinity, elevated pressure, and ecological diversity of marine environments have driven the evolution of unique secondary metabolites in marine organisms [19]. Statistical evidence indicates that marine natural product chemical structural diversity substantially exceeds terrestrial sources, frequently incorporating rare heterocycles and stereochemical features [46]. In recent years, multiple marine natural product-derived anticancer drugs have garnered regulatory approval, including trabectedin sourced from sea squirts and eribulin derived from sea hares [47,48].

Marine natural products confer additional advantages including comparatively attenuated cytotoxicity and enhanced selectivity windows. Numerous marine-sourced compounds exert effects through modulation of specific signaling pathways rather than direct DNA disruption, thereby reducing normal cell damage [49]. CMNPD30506, as a kinase inhibitor candidate, operates through mechanisms distinct from conventional cytotoxic agents, potentially offering superior therapeutic indices. Furthermore, marine natural products typically exhibit favorable drug-likeness, as their evolutionary processes necessitate interactions with biological macromolecules, conferring tremendous potential in pharmaceutical domains [50,51]. Our study, through rigorous Lipinski rule and QED scoring filtration, ensured candidate compounds possess promising pharmacokinetic potential.

This investigation integrated multiple cutting-edge AI and computational tools, representing technological advances in drug discovery. The UniSite deep learning model for pocket prediction demonstrates superior accuracy in identifying cryptic and allosteric pockets compared to conventional geometric algorithms. DiffDock’s diffusion model-based molecular docking methodology transcends sampling efficiency bottlenecks of traditional docking tools, achieving markedly improved success rates in blind docking tasks relative to classical tools such as AutoDock Vina and Glide. These AI tools enabled screening of nearly hundred-thousand-scale compound libraries at reasonable computational costs—tasks requiring supercomputing clusters a decade ago.

However, AI methodologies harbor limitations. The black-box nature of deep learning models constrains interpretability of predictive outcomes [52]. Accordingly, we adopted multilayer validation strategies: DiffDock screening followed by conventional QVina2 docking validation, subsequently corroborated through MD simulations and MM-GBSA calculations providing physicochemical confirmations. This hybrid AI screening plus physical validation workflow ensures both efficiency and reliability. Moreover, a critical limitation requiring explicit acknowledgment is the systematic target bias observed in our DiffDock virtual screening campaign. As noted in our results, very few compounds from either the COCONUT or CMNPD libraries achieved confidence scores exceeding 0 for VEGFR2, whereas substantially more compounds achieved high confidence scores (>0) for c-Met. This asymmetric score distribution has important implications for our “dual-target” selection strategy. Several factors may contribute to this bias: DiffDock’s training data composition may include more c-Met or c-Met-like kinase complexes, leading to better-calibrated confidence scores for this target; VEGFR2’s ATP-binding pocket may present greater pose prediction challenges due to structural flexibility or pocket geometry; marine natural product scaffolds may exhibit closer structural similarity to known c-Met ligands than VEGFR2 inhibitors, affecting confidence score distributions.

Despite furnishing compelling computational evidence for CMNPD30506 as a putative dual-target lead candidate through multiscale methodologies, several limitations warrant resolution in subsequent investigations. Firstly, all conclusions derive from computational predictions lacking experimental validation. Future steps necessitate in vitro experimental assessments of antiproliferative activities and pathway blockade effects. Additionally, a critical limitation of this study is the absence of kinase selectivity profiling. The computational analyses presented evaluate binding only to c-Met and VEGFR2; therefore, we cannot assess off-target kinase binding, which is essential for therapeutic development. Future investigations should prioritize kinome-wide computational docking screens against structurally characterized kinases, followed by experimental validation using kinase activity profiling platforms. Such studies would identify potential off-target kinases and guide structure-activity relationship optimization to improve selectivity margins [53]. Additionally, comparative structural analysis of CMNPD30506 binding modes across different kinase families could elucidate molecular determinants of selectivity and inform rational design strategies. It is important to emphasize that the computational binding metrics reported here have not been benchmarked against clinically validated c-Met/VEGFR2 dual inhibitors such as cabozantinib under identical computational protocols. Therefore, while our results suggest that CMNPD30506 forms stable computational binding modes with both targets, we cannot conclude whether its predicted affinity represents strong, moderate, or weak performance relative to known active compounds. Future studies incorporating reference inhibitors evaluated under the same workflow would provide essential context for interpreting these findings.

Secondly, pharmacokinetic and toxicity predictions require refinement. While we conducted Lipinski and QED scoring assessments, key ADMET properties including oral bioavailability, blood-brain barrier permeability, and hERG cardiotoxicity demand more nuanced predictions and experimental validations. DFT-revealed potential metabolic susceptibility also necessitates confirmation through hepatic microsomal stability assays and cytochrome P450 inhibition/induction experiments. Furthermore, natural product sourcing and scale-up production present practical application challenges, potentially requiring total synthesis or semisynthetic strategies.

It is important to emphasize that structural stability observed in molecular dynamics simulations reflects the thermodynamic favorability of the predicted binding mode under the applied force field and simulation conditions, and does not directly imply biological inhibitory activity, kinase selectivity, or therapeutic efficacy. These findings should be interpreted as evidence supporting the feasibility of the proposed binding mode rather than as a surrogate for experimental potency measurements. Additionally, only a single 200 ns trajectory was performed for each protein-ligand system. While the RMSD profiles suggest conformational stabilization within the simulation timescale, we acknowledge that single-trajectory analyses are subject to sampling limitations and may not fully capture the conformational heterogeneity of the binding site. An important methodological limitation is the absence of docking protocol validation through redocking of co-crystallized ligands with RMSD-based accuracy assessment for the specific VEGFR2 and c-Met systems used in this study. While the DiffDock platform has been benchmarked extensively in its original publication, and our use of orthogonal docking validation (QVina2) plus extended MD simulations provides some confidence in binding mode reliability, system-specific redocking validation would further strengthen these conclusions. Future applications of this workflow should incorporate such validation protocols.

Finally, from translational medicine perspectives, future endeavors require in vivo efficacy validation. Establishing HCC patient-derived xenografts or genetically engineered mouse models to evaluate CMNPD30506 monotherapy or combination therapies with immune checkpoint inhibitors’ antitumor effects. Considering HCC’s pronounced heterogeneity and differential molecular subtype sensitivities to targeted therapies, biomarker investigations prior to clinical translation will facilitate patient stratification and precision therapeutic strategy formulation.

## 5. Conclusions

In summary, through integration of deep learning virtual screening, molecular dynamics simulations, and quantum chemical calculations, this study successfully identified the marine natural product CMNPD30506 from large-scale natural product libraries as a lead compound exhibiting dual c-Met/VEGFR2 inhibitory potential. Multilevel computational validations substantiate that this compound forms stable binding complexes with both targets, demonstrating balanced dual-target affinity and favorable thermodynamic characteristics. This discovery not only furnishes novel candidate molecules for dual-target HCC therapeutic development but also exemplifies the application value of AI-driven drug discovery paradigms in multi-target pharmaceutical design. Subsequent experimental validations and structural optimization investigations will further advance this lead compound toward clinical candidate drug translation.

## Figures and Tables

**Table 1:** Physicochemical properties and drug-likeness profiles of the candidate compounds identified from the integrated virtual screening.

Source	Compound ID	Molecular Formula	InChIKey	MW	AlogP	RB	HBA	HBD	TPSA	Ar. Rings	HA	QED
COCONUT	CNP0376925.2	C19H21NS	PHTUQLWOUWZIMZ-UHFFFAOYSA-N	295.14	4.68	3	2	0	3.24	2	21	0.81
COCONUT	CNP0073361.1	C15H16O5	OCAHDNBHANRMSH-KEMSKJLVSA-N	276.10	1.94	1	5	2	75.99	1	20	0.82
CMNPD	CMNPD30538	C14H16N2O	NHSHMCBGMNXSIT-UHFFFAOYSA-N	228.30	2.73	3	1	2	58.88	2	17	0.78
CMNPD	CMNPD30506	C10H10O4	ULGWVUDHZXZJSA-VIFPVBQESA-N	194.19	1.72	1	4	2	66.76	1	14	0.53

**Table 2:** Binding affinities of the top-ranked candidate compounds against VEGFR2 and c-Met.

Source	Compound ID	VEGFR2 BE	c-Met BE	Mean BE
COCONUT	CNP0376925.2	−0.7	1.4	0.4
COCONUT	CNP0073361.1	−1.0	−0.1	−0.6
CMNPD	CMNPD30538	−6.1	−6.6	−6.4
CMNPD	CMNPD30506	−5.9	−7.3	−6.6

BE, Binding Energy (kcal/mol).

## Data Availability

All data supporting the findings of this study are available within the article and its Supplementary Materials. The protein structures used in this study were retrieved from the Protein Data Bank (https://www.rcsb.org/; accessed on 22 February 2026; PDB IDs: 2XIR for VEGFR2 and 4R1V for c-Met). The natural product compound libraries were obtained from the COCONUT database (https://coconut.naturalproducts.net/; accessed on 8 December 2025) and the CMNPD database (https://cmnpd.org/; accessed on 13 December 2025).

## Supplementary Materials

The following supporting information can be downloaded at: https://www.mdpi.com/article/10.3390/cimb48090902/s1.

## Abbreviations

The following abbreviations are used in this manuscript:

ADMET	Absorption, Distribution, Metabolism, Excretion, and Toxicity
AI	Artificial intelligence
AIDD	Artificial intelligence in drug discovery
BE	Binding Energy
CADD	Computer-aided drug design
CMNPD	Comprehensive Marine Natural Products Database
COCONUT	COlleCtion of Open Natural prodUcTs
DFT	Density functional theory
EMT	Epithelial-mesenchymal transition
FDA	Food and Drug Administration
FMO	Frontier molecular orbital
HCC	Hepatocellular carcinoma
HGF	Hepatocyte growth factor
HIF-1α	Hypoxia-inducible factor-1α
HOMO	Highest occupied molecular orbital
LUMO	Lowest unoccupied molecular orbital
MESP	Molecular electrostatic potential
MM-GBSA	Molecular Mechanics/Generalized Born Surface Area
NBP	3-n-butylphthalide
NPT	Constant pressure and temperature
PCA	Principal component analysis
PDB	Protein Data Bank
PLIP	Protein-Ligand Interaction Profiler
PME	Particle Mesh Ewald
QED	Quantitative Estimate of Drug-likeness
RESP	Restrained electrostatic potential
Rg	Radius of gyration
RMSD	Root mean square deviation
RMSF	Root mean square fluctuation
RO5	Rule of Five (Lipinski’s Rule)
RTK	Receptor tyrosine kinase
TKIs	Tyrosine kinase inhibitors
VEGFR2	Vascular endothelial growth factor receptor 2
